# Expression of VISTA on T cells in patients with cancer colon

**DOI:** 10.1186/s43046-025-00287-x

**Published:** 2025-05-23

**Authors:** Amira Gamal El Dein Badary, Rania Bakry, Khalid Rezk, Maged AF Amine, Asmaa M.Zahran

**Affiliations:** 1 Molecular Biology Research & Studies Institute, Assiut, Egypt; 2 South Egypt Cancer Institute, Clinical Pathology Department, Assuit, Egypt; 3 South Egypt Cancer Institute, Surgical Oncology Department, Assiut, Egypt; 4 South Egypt Cancer Institute, Medical Oncology and Hematological malignancies, Assiut, Egypt

**Keywords:** VISTA, T cell subsets, Colorectal cancer (CRC)

## Abstract

Colorectal cancer (CRC) is one of the most common tumors in the world. A recent area of study for the treatment of patients with solid tumors is anti-tumor immunity. PD-1/PD-L1 inhibitors were beneficial for cancer patients with multiple tumor types. However, their efficacy for CRC is low. Thus, there is an urgent need to explore additional co-inhibitory tools such as VISTA for CRC treatment.

**Objective**

The current study aimed to evaluate expression of VISTA on T cell subsets in patients with CRC and its correlation with other prognostic markers.

**Patients and methods**

The study included 31 patients with CRC and 25 healthy controls. All participants were subjected to full history taking, clinical examination, routine laboratory investigations, and flow cytometric detection of VISTA expression on T cell subsets on peripheral blood (PB). In addition to detection of VISTA expression on T cell subsets on tissue samples of both malignant CRC and normal colon tissue of the CRC patients.

**Results**

In the peripheral blood, the expression of VISTA on CD4^+^ T helper and CD8^+^ T cytotoxic cells was significantly higher in CRC patients than the normal controls. There was no significant difference in VISTA expression on double positive T cells (CD4^+^CD8^+^) between the CRC patients and normal controls. In tissue samples, expression of VISTA on CD4 + T helper, CD8 + T cytotoxic, and double positive T (CD4 + CD8 +) cells in the malignant tissue of CRC patients was significantly higher than that in normal colonic tissue. Also, in CRC patients, the expression of VISTA on CD4^+^ T helper, CD8^+^ T cytotoxic, and double positive T cells in both malignant CRC tissue and normal colonic tissue was significantly higher than its expression PB.

**Conclusion**

The higher expression of VISTA in CRC patients than the healthy controls and its higher levels in malignant CRC tissue and normal colonic tissue than PB of CRC patients suggest the role VISTA in the pathogenesis of CRC.

## Introduction

Colorectal cancer (CRC) is one of the most common tumors in the world. There are more than 1.2 million cases of CRC diagnosed each year; it is the third most common cause of cancer that affects humans [1, 2]. According to Sung et al. [3], CRC accounts for 3.8% of cancer-related deaths in Egypt and has an incidence rate of roughly 6.1% of cases. With 35% of 1600 Egyptian CRC patients being under 40, Egypt has one of the highest rates of early CRC worldwide. According to a study, Egyptian individuals with colorectal cancer (CRC) under the age of 30 have a threefold higher chance of passing away within 5 years than those with CRC over 50 [4].

Several studies reported that the immune system plays a role in tumor suppression, tumor growth, and survival [5]. Cytotoxic innate and adaptive immune cells can regulate the growth of tumors; nevertheless, when the tumor progresses from neoplastic tissue to clinically identifiable tumors, cancer cells develop distinct defense mechanisms that resemble peripheral immunological tolerance to evade tumoricidal attack [6].

Immune checkpoints are sets of pathways that regulate and control the durability of the immune response. There are two kinds of signals: co-stimulatory immune checkpoint, stimulating immune progress, such as CD28, ICOS, and CD137, and co-inhibitory immune checkpoint, inhibiting immune progress, such as programmed death- 1 (PD- 1), cytotoxic T lymphocyte associated molecule- 4 (CTLA- 4), T cell immunoglobulin mucindomain-containing- 3 (Tim- 3), T cell lymphocyte activation gene- 3 (LAG- 3), and B and T lymphocyte attenuator (BTLA), plays a critical role in reducing T cell activation and promoting T cell exhaustion and V-domain immunoglobulin suppressor of T cell activation (VISTA) [7–10].

V-domain immunoglobulin suppressor of T cell activation (VISTA) also known as PD- 1H, B7-H5, VSIR, and c10orf54 is a novel checkpoint regulator. A type I transmembrane protein is known as a unique B7 family member expressed on a variety of immune cells (ICs) including lymphocytes, dendritic cells, and macrophages, and participates in cell activation and functional regulation and tumor-infiltrating lymphocytes (TILs) has become a current focus of research [11–14]. VISTA suppresses T cell activation and contributes to the immune evasion of tumors. While previous reports have highlighted the significance of this protein in a number of malignancies [15–26]*,* rare of them are known about its expression on T cell subsets in CRC.

The role of VISTA in T cell exhaustion and immune suppression makes it an attractive target for cancer immunotherapy, particularly in solid tumors like CRC. While still in early stages of clinical development, VISTA inhibitors are being tested, and there is growing interest in combining them with other checkpoint inhibitors (e.g., PD- 1 or CTLA- 4 inhibitors) to improve immune responses against cancer. As research progresses, VISTA may become an important therapeutic target, especially for cancers that are less responsive to current immunotherapies.

## Aim of the study

Evaluate expression of VISTA in T cell in patients with cancer colon and correlated with other prognostic markers.

## Subjects and methods

This was a hospital-based case–control study that was conducted at South Egypt Cancer Institute (SECI), Assiut University from February 2023 to January 2024. The study included 31 patients with CRC who presented and 25 normal controls with age and sex matched with patients and they are selected from people who do not suffer from any immune diseases.

The Inclusion criteria: Patients diagnosed CRC according to the world health organization (WHO) [27]; patients selected from those who underwent upfront surgery; patients with age more than 18 years.

The exclusion criteria included: Patients with previous treatment history for CRC, patients suffered from another type of cancer in addition to CRC, patients with an infectious disease, chronic liver, or renal disease, patients with age less than 18 years, and patients unwilling to participate in the study.

### Method

All patients have been subjected to full medical history taking and thorough clinical examination. We also performed routine laboratory investigations that included complete blood count (CBC) liver function tests, kidney function tests, and flow cytometric detection of VISTA expression on T cells on PB of patients and normal controls. In addition to, CEA and CA19.9 according to routine methods and flow cytometric detection of VISTA expression on T cells on PB, normal tissue, and malignant tissue of patients only.

### Flow cytometric detection of the frequency of T cell subsets and their VISTA expression

#### Blood samples for flow cytometry

A peripheral venous blood sample (3 ml) was collected from each patient before surgery and control individual in EDTA tubes labeled with the subject’s name, sex, age, the date of collection, and patient number in the SECI for analyzing of T cells subsets and their VISTA expression by flow cytometry.

#### Tissue sample from CRC patients

From each patient were included in this study, a piece of CRC malignant tissue and apiece after safety margin as normal tissue (5 cm away from the visible tumor mass) was collected within surgery. Collection of tissue samples was performed in operation rooms in SECI. The tissue samples were placed in separated cups containing saline and labeled with the patient’s name, sex, age, date of collection, and patient number in the SECI.

#### Sample staining, acquisition, and analysis of PB

T cell subsets and their VISTA expression were detected in peripheral blood samples of patients and healthy controls, and in malignant CRC tissue and normal colonic tissue of CRC patients

To detect T cell subsets and their VISTA expression in blood samples, 100 µl blood samples mixed with 10 µl from each phycoerythrin (PE-Cy7) conjugated anti-VISTA (Invitrogen, USA), peridinin-chlorophyll-protein (Percp) conjugated anti-CD4 (BD Bioscience, USA), and allophycocyanin (APC) conjugated anti-CD8 (Beckman coulter, France). The mixture was then incubated for 15 min in the dark at room temperature. After incubation, red blood cells were lysed with BD FACS Lysing solution 10x (Cat. No. 349202) (BD Biosciences, USA) and the cells were then washed with magnesium and calcium free phosphate buffer saline solution (PBS) (Cat. No. 17 - 516 F) (Lonza, BioWhittaker®, USA) and resuspended in PBS. An isotype negative control was run with each sample. Samples were then analyzed by a flow cytometer FACS Canto II (BD Bioscience-San Jose, CA, USA, serial no. V33896201978; 3 lasers, 8 color) or Navios EX (Beckman Coulter life science, serial no.BF22S40; 3 lasers, 10 color). A minimum of 50.000 events were collected from each sample. Data were analyzed using FACS DIVA software version 8.0.1 on FACS Canto II and Kaluza C software on Navios EX 3 according to the manufacturer’s instructions. The lymphocyte population was identified by their size and granularity characteristic of cells using forward scatter and side scatter dot plot. Then the expression of CD4^+^ and CD8^+^ was detected on lymphocytes population to detected CD4^+^ T helper, CD8^+^ T cytotoxic, and CD4^+^CD8^+^ double positive T cells which were then gated for further detection of their VISTA expression.

#### Sample staining, acquisition, and analysis of tissue samples

To detect T cell subsets and their VISTA expression in in malignant CRC tissue and normal colonic tissue of CRC patients, the fresh tissue was transferred to a petri dish and washed with 3 ml PBS to remove any trace of salt solution and then begin the mechanical grinding of tissue by using sharp scalpel. After proper grinding of tissue, suspension (PBS with cells from tissue) was filtered by 40-μL falcon mish installed on 50-ml polystyrene round-bottom falcon tubes to be sure that there is no tissue particles or fibers. After filtration, the cell suspension was transferred into 5-ml polystyrene round-bottom falcon tubes. Tubes were centrifuged at 2500 rpm for 1 min and the supernatant was discarded. Then the previous procedure steps of peripheral blood were performed on filtered suspension.

## Statistical analysis

Based on determining the main outcome variable, the estimated minimum required sample size is 31 patients and 25 controls. The sample was calculated using G*power software 3.1.9.2. Data were verified, coded by the researcher, and analyzed using the Statistical Package for the Social Sciences (SPSS) version 29.0 (SPSS- IBM Inc., Chicago, IL, USA) and GraphPad Prism (version 10.1.1, GraphPad software, LLC). Test of normality was done for deciding the measures of central tendency and statistical methods for data analysis by using Kolmogorov–Smirnov test and Shapiro–Wilk test. Categorical variables were described by number and percent, whereas continuous variables were described by the mean and standard error (SE). Mann–Whitney *U* test was used to analyze data for two independent samples, and the Kruskal–Wallis test was used to compare different groups more than two. Spearman correlation was applied to evaluate the association between variables. *p*-value was ≤ 0.05 was considered statistically significant.

## Results

### The demographic data, clinical manifestations, and pathological finding as shown in Table 1

Of the total 31 patients, there were 22 (71%) males and 9 (29%) females with the mean age of 52.71 ± 2.390 and range of 30–81 years. 21 (67.7%) were nonsmoker and 10 (32.3%) smokers. Abdominal pain was the most common presented manifestation, followed by change in bowel habitat (diarrhea or constipation), then bleeding and finally weight loss in patients. According to pathological analysis patients in the study, adenocarcinoma represented 23 (74.19%) patients and Mucinous adenocarcinoma represented 8 (25.8%) patients. 64.56% of the patients had tumor in the left side of the colon and only 35.48% patients right side of the colon, as shown in Table 1.

**Table 1:** Demographic, clinicopathological, and pathological characteristics of 31 CRC patients evaluated for the Expression of VISTA on T cells and tissue cells

**Variables**		**Colorectal cancer patients (*N*** **= 31)**	
		***n***	**%**
Age	Mean	52.71 ± 2.390	
	Range	30–81	
Gender	Male	22	71%
	Female	9	29%
Smoking	Yes	21	67.7%
	No	10	32.3%
Clinical features of CRC patients			
Abdominal pain		15	48.39%
Bleeding		5	16.3%
Change in bowel habit		9	29.03%
Weight loss		2	6.45%
Pathological finding			
Adenocarcinoma		23	74.19%
Mucinous adenocarcinoma		8	25.8%
Anatomical location			
RT colon cancer		11	35.48%
LT colon cancer		20	64.56%

*RT* colon cancer right colon cancer, *LT* colon cancer left colon cancer. *N* number of cases, % percentage

### Classification of colorectal cancer patients according to T, N, M, staging and grading of cancer

As regards TNM staging, 21 patients (67.6%) at “T3” followed by 8 patients (25.8%) at “T2,” and 2 patients (6.5%) at “T4.” Both “N0” and “N1” were detected in 13 patients for each (41.9%), while 5 patients (16.13%) exhibited “N2.” Concerning metastasis, 24 patients (77.4%) were diagnosed with nonmetastatic disease “M0” and 7 patients (22.58%) were diagnosed with metastatic disease “M1.” The most common AJCC stage of CRC presented in the study group was stage (III) which detected in 15 patients (48.39%) followed by stage (II) in 8 patients (25.8%), stage (I) in 6 patients (19.35%), and finally stage (IV) in 2 patients (6.4%). All patients in our study were classified as grade 2, as shown in Table 2.

**Table 2:** TNM staging of 31 CRC patients evaluated for the Expression of VISTA on T cells and tissue cells

**Variables**		**Colorectal cancer patients (*N*** **= 31)**	
		***n***	**%**
T (%)	T2	8	25.8%
	T3	21	67.7%
	T4	2	6.5%
N (%)	N0	13	41.9%
	N1	13	41.9%
	N2	5	16.13%
M (%)	M0	24	77.4%
	M1	7	22.58%
TNM (%)	I	6	19.35%
	II	8	25.8%
	III	15	48.39%
	IV	2	6.4%
Tumor grade (%)	G2	31	100%

Tumor’s expanse (size) (*T*), nearby lymph nodes (*N*), the spread (metastasis) to new areas (*M*), I: stage 1, II: stage 2, III: stage 3, IV: stage 4. N number of cases, % percentage

### Laboratory data of patients with CRC and normal controls

There was significantly increase in total leukocytes count (TLC), neutrophils, monocytes, platelets count, neutrophil/lymphocyte ratio (NLR), and platelet/lymphocyte ratio (PLR) on CRC patients than the normal controls. However, lymphocyte count, lymphocytes/monocytes ratio (LMR), red blood cells (RBCs), and hemoglobin (HB) level were significantly decrease in CRC patients than the normal controls. There were significantly increase in the levels of urea, aspartate transaminase (AST), alkaline phosphatase (ALP), and phosphorus and lactate dehydrogenase (LDH) in CRC patients than the normal controls. There was significantly decrease in total protein and albumin in patients than the normal controls. CRC patients in study group have CEA with mean (5.82 ± 4.14) and median (1.7), where the normal range in nonsmoker is up to 2.5 ng/ml and in smoker patients up to 5 ng/ml, and CA19.9 with mean (18.820 ± 3.47) and median (15.0), where the normal range is up to 37 U/ml, as shown in Table 3.

**Table 3:** Laboratory data of 31 CRC patients and 25 normal controls evaluated for the Expression of VISTA on T cells and tissue cells

Variable	CRC patients (*n* = 31)	Controls (*n* = 25)	*p*-value
**Hematological findings**			
RBCs (3.8–5.2 × 10^6^/µL)	4.4416 ± 0.13	5.61 ± 0.05	0.035
HB (11.5–15.5 g/dl)	11.135 ± 0.28	13.78 ± 0.09	0.04
TLC (4.5–11 × 10^3^/µL)	10.735 ± 1.4446	6.344 ± 0.3689	0.002
Neutrophil’s count %	77.113 ± 2.1557	59.672 ± 2.4642	0.0001
Neutrophils absolute (2–7 × 10^3^/µL)	7.4087 ± 0.73645	4.7996 ± 0.63698	0.003
Lymphocyte’s count %	14.850 ± 1.59997	26.4160 ± 1.83163	0.0001
Lymphocytes absolute (1.5–4 × 10^3^/µL)	1.2500 ± 0.13638	2.4048 ± 0.17762	0.0001
Eosinophil’s count %	1.184 ± 0.3432	0.952 ± 0.1439	0.235
Eosinophils absolute (up to 0.1)	0.0748 ± 0.01824	0.0776 ± 0.01257	0.439
Monocyte’s count %	6.442 ± 0.4461	1.760 ± 0.1661	0.001
Monocytes absolute (0.2–1 × 10^3^/µL)	0.5455 ± 0.04711	0.1344 ± 0.01567	0.001
Platelet (150–450 × 10^3^/µL)	301.26 ± 21.309	233.68 ± 7.862	0.015
Renal functions			
Blood urea (mg/dL)	26.56 ± 1.206	20.39 ± 1.422	0.001
Serum creatinine (mg/dL)	0.7516 ± 0.039	0.7324 ± 0.035	0.739
Liver functions			
AST (U/L)	19.65 ± 2.057	11.96 ± 0.689	0.001
ALT (U/L)	17.65 ± 2.676	11.32 ± 0.596	0.105
ALP (U/L)	84.94 ± 5.546	52.28 ± 2.28	< 0.001
Total bilirubin (mg/dl)	0.552 ± 0.0595	0.404 ± 0.0261	0.061
Direct bilirubin (mg/dl)	0.242 ± 0.0488	0.164 ± 0.0140	0.214
In direct bilirubin	0.310 ± 0.0264	0.240 ± 0.0173	0.080
Total protein (g/L)	60.42 ± 1.303	63.72 ± 5.08	0.016
Albumin (g/L)	35.03 ± 1.363	43.24 ± 1.044	0.001
Globulin (g/L)	25.35 ± 1.114	26.08 ± 0.868	0.514
LDH (U/l)	194.42 ± 10.310	89.08 ± 4.264	0.001
Tumur marker			
CEA (non-smoker: up to 2.5 ng/ml) (smoker: up to 5 ng/ml)	5.82 ± 4.14 (1.7)	–––––	
CA19.9 (up to 37 U/ml)	18.820 ± 3.47 (15.0)	–––––	
Ratio			
NLR	8.26 ± 1.186	0.890 ± 0.178	< 0.001
PLR	30.077 ± 5.40	3.97 ± 0.794	< 0.001
LMR	2.41 ± 0.24	17.66 ± 1.6	< 0.001

*CRC* colorectal cancer, *RBG* random blood glucose, *TLC* total leukocytes count, *AST* aspartate transaminase, *ALT* alanine transaminase, *ALP* alkaline phosphatase, *LDH* lactate dehydrogenase, *INR* international normalized ratio, *CEA* carcinoembryonic antigen, CA19.9 cancer antigen 19.9, NLR neutrophil/lymphocyte ratio, *LMR* lymphocyte/monocyte ratio, and *PLR* platelet/lymphocyte ratio. Quantitative data are presented as mean ± standard error (SE) and (median). Mann–Whitney *U* test, *p*-value is significant at < 0.05 (bold). *p* CRC patients vs. normal controls

### T cell subsets and their VISTA expression in PB of CRC patients and the normal controls

**Fig. 1:**
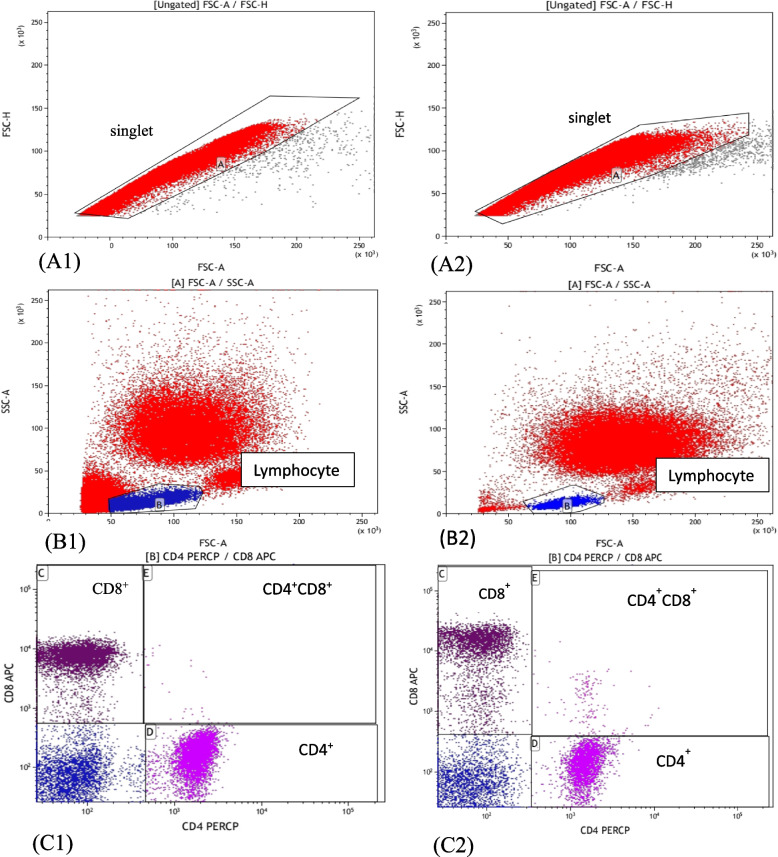
(A1, A2) Singlet cells in PB of the normal controls and CRC patients, respectively. (B1, B2) Forward scatter and side scatter dot plots to detect lymphocytes cells in PB of the normal controls and CRC patients, respectively. (C1, C2) T cells subsets (CD4^+^ T helper (gate D), CD8^+^ T cytotoxic (gate C), and CD4^+^CD8^+^ (gate E) double positive T cells) in PB of the normal controls and CRC patients, respectively

**Fig. 2:**
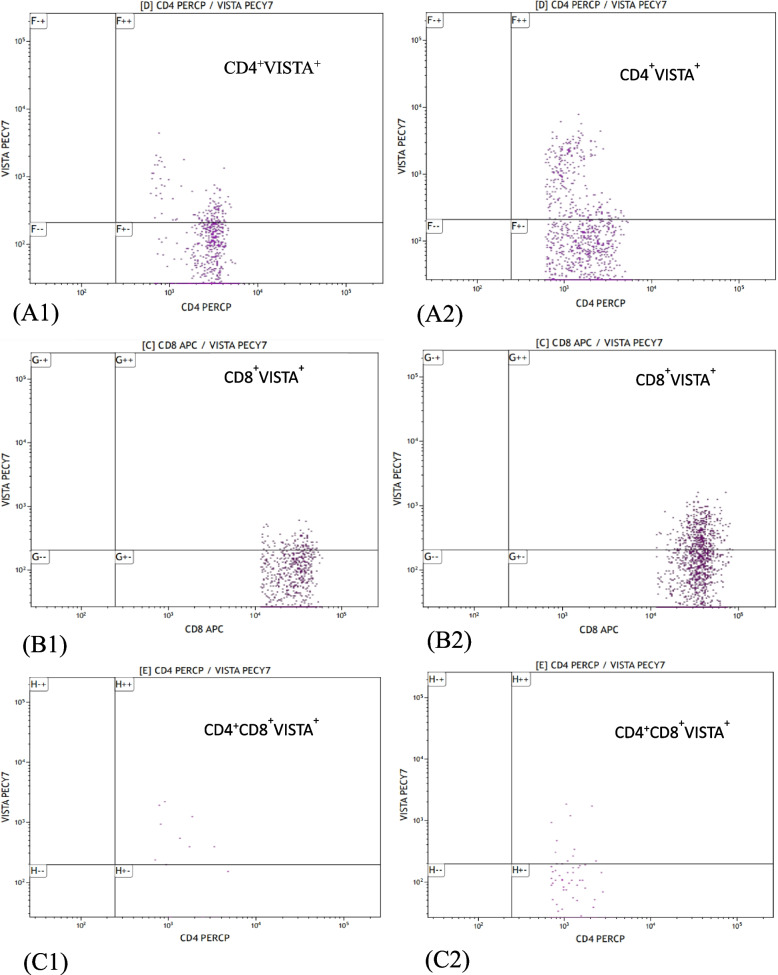
Expression of VISTA on the following: (A1, A2) CD4 + on PB of the normal controls and CRC patients, respectively. (B1, B2) CD8 + on PB of the normal controls and CRC patients, respectively. (C1, C2) CD4 + CD8 + on PB of the normal controls and CRC patients, respectively

**Fig. 3:**
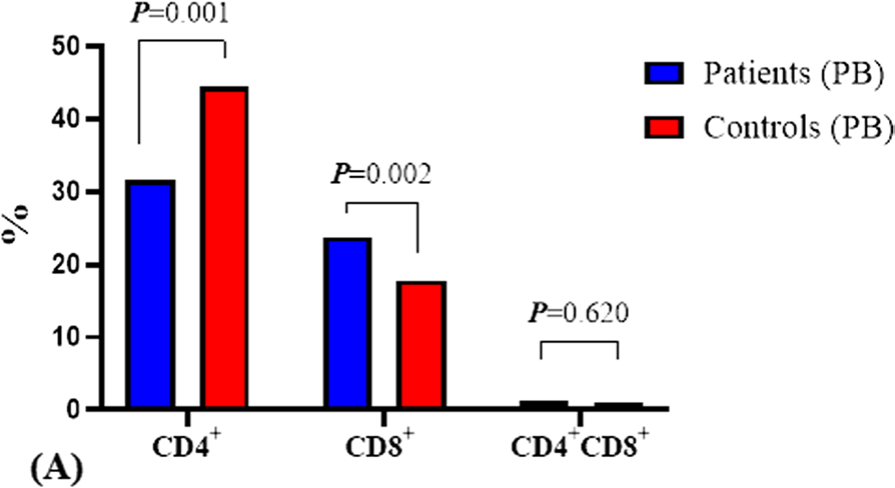
Different T cell subsets (T helper (CD4 +), T cytotoxic (CD8 +), and double positive T cells (CD4 + CD8 +) in CRC patients and normal control PB

**Fig. 4:**
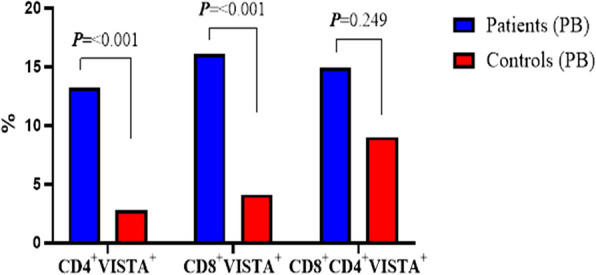
Different expression of VISTA on T helper cells, T cytotoxic cells, and double positive T cells in PB of CRC patients and the normal controls

The level of CD4^+^ T helper cells in CRC patients was significantly lower than in the normal controls. The level of CD8^+^ T cytotoxic cells in the CRC patients was significantly higher than that in the normal control cases, while there was no significant difference in the level of double positive T cells (CD4^+^CD8^+^) in the CRC patients and the normal control. The expression of VISTA on CD4^+^ T helper and CD8^+^ T cytotoxic were significantly higher in CRC patients PB than the normal controls. Also, VISTA expression on double positive T cells (CD4^+^CD8^+^) was not significantly different between CRC patients and the normal controls, as shown in Table 4 and Figs. 1, 2, 3 and 4.

**Table 4:** T cell subsets and their VISTA expression in PB of 31 CRC patients and 25 normal controls

Variables	CRC patients (*n* = 31)	Normal control (*n* = 25)	*p*-value
T helper (CD4^+^) cells (%)	31.55 ± 1.27	44.62 ± 1.04	0.001
CD4^+^VISTA^+^cells (%)	13.23 ± 2.62	2.82 ± 0.24	< 0.001
T cytotoxic (CD8^+^) cells (%)	23.78 ± 1.32	17.84 ± 0.798	0.002
CD8^+^VISTA^+^cells (%)	16.10 ± 2.25	4.12 ± 0.23	< 0.001
CD4^+^CD8^+^ T cells (%)	1.16 ± 0.12	1.0 ± 0.1	0.620
CD4^+^CD8^+^VISTA^+^cells (%)	14.92 ± 2.34	9.00 ± 0.76	0.249

Results are expressed as mean ± standard error of mean Mann–Whitney *U* test, *p*-value is significant at < 0.05

*p-value*, CRC patients vs. normal controls

**Table 5:** Determine the localized expression pattern of VISTA on T cell subsets in PB of CRC patients and the normal controls

Variables	CD4^+^VISTA^+^	CD8^+^VISTA^+^	CD4^+^CD8^+^VISTA^+^	*p*-value
Patients	13.23 ± 2.62	16.10 ± 2.25	13.15 ± 2.34	0.910
Normal controls	2.82 ± 0.24	4.12 ± 0.23	7.93 ± 0.49	< 0.001

Results are expressed as mean± standard error of mean

Kruskal–Wallis test, *p*-value is significant at < 0.05

As shown in Table 5, expression of VISTA on PB of CRC patients was not significantly different between CD4^+^T helper cells, CD8^+^T cytotoxic cells, and CD4^+^CD8^+^ double positive cells. However, expression of VISTA on PB on the normal controls was significantly higher on ((CD4^+^CD8^+^) double positive T cells than on (CD4^+^) T-helper cells, and on (CD8^+^) cytotoxic T cells.

#### Relation between peripheral blood T cells subsets and their VISTA expression and CRC site

T cell subsets and their expression of VISTA on PB of CRC patients show no significant association with CRC site.

#### Relation between CRC stages and T cells subsets and their VISTA expression in PB

T cells subsets and their expression of VISTA on PB of CRC patients show no significant association with T, N, M and stages of CRC patients.

#### Relation between CRC prognostic markers and T cells subsets and their VISTA expression in PB

As shown on Table 6, there was negative significant correlation between the frequency of CD4^+^ T helper cell and HB. In addition to, positive correlation between the expression of VISTA on CD4^+^ T helper cell with HB, WBCs, and platelet count, and positive correlation between the expression of VISTA on CD8^+^ T helper cell with platelet count and CEA.

There was no significant correlation between T cell subsets and their VISTA expression and age, NLR, PLR, urea, LDH, ALP, AST, ALT, total protein, albumin, and CA19.9.

### T cell subsets and their VISTA expression in malignant tissue and normal colonic tissue of CRC patients

**Fig. 5:**
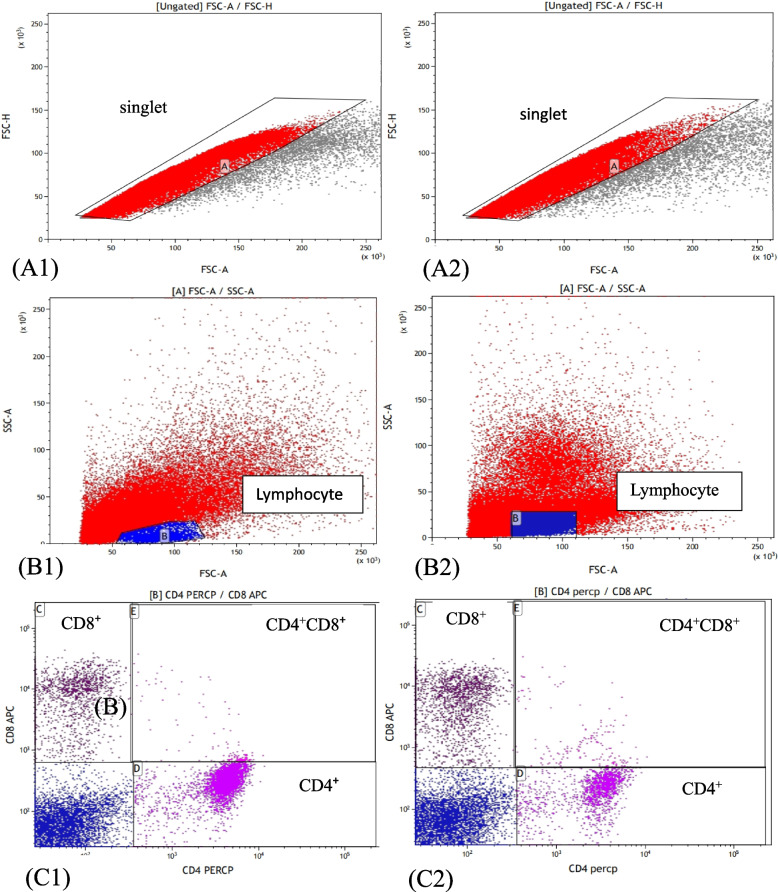
Dot plot shows the following: A1 and A2 represent singlet cells in normal and malignant tissue, respectively. B1, B2 Forward scatter and side scatter dot plots to detect lymphocyte cells in normal and malignant tissue, respectively. C1, C2 T cells subsets (CD4^+^ T helper, CD8^+^ T cytotoxic, and CD4^+^CD8^+^ double positive T cells) in normal and malignant tissue, respectively

**Fig. 6:**
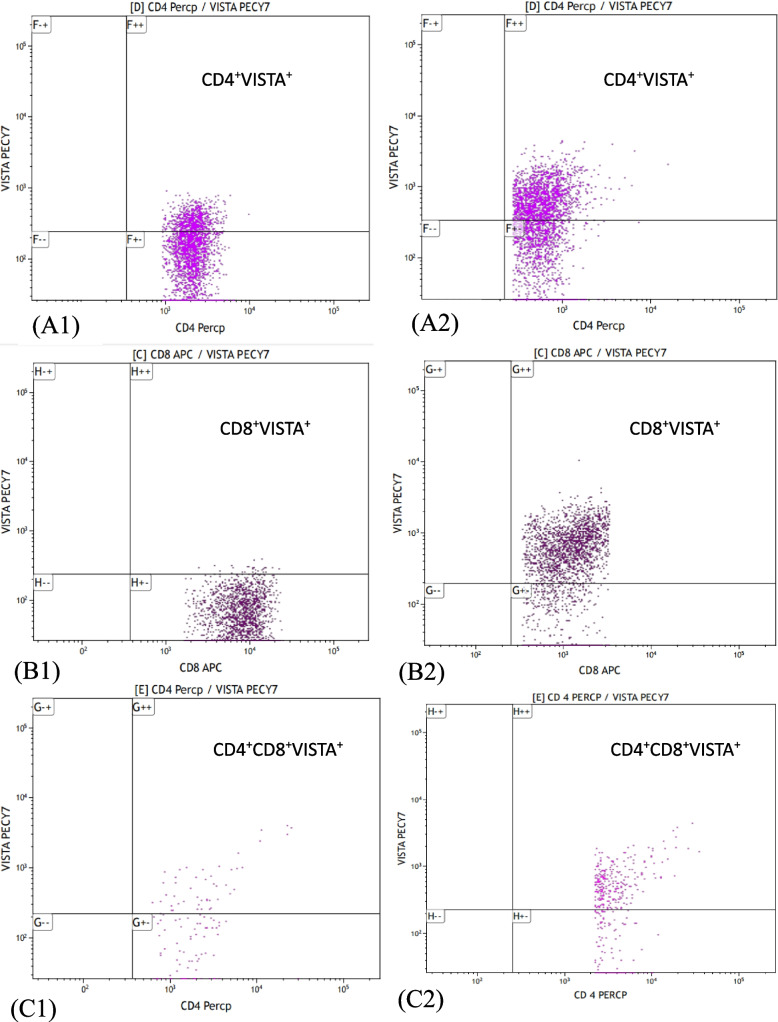
Expression of VISTA on T cell subsets: on CD4^+^T helper cells (A1 and A2), on CD8^+^T cytotoxic cells (B1 and B2), and on CD4^+^CD8.^+^ double positive T cells (C1 and C2)

**Fig. 7:**
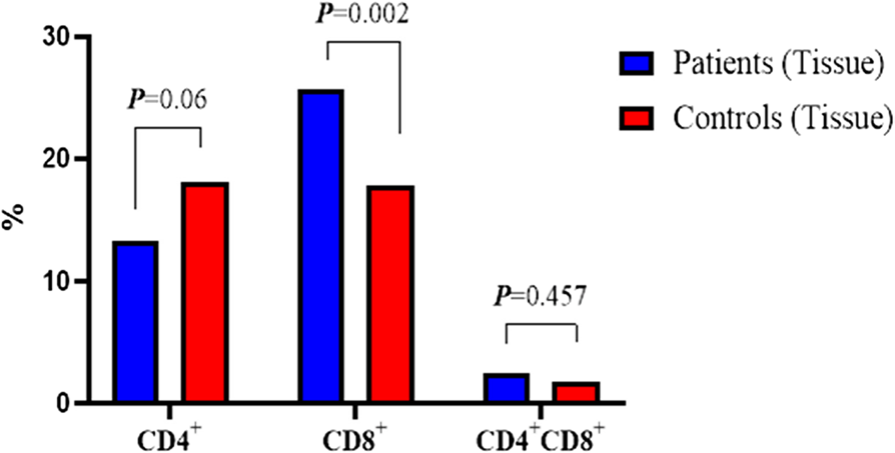
Frequency of T cell subsets on malignant tissue and normal tissue of CRC patients

**Fig. 8:**
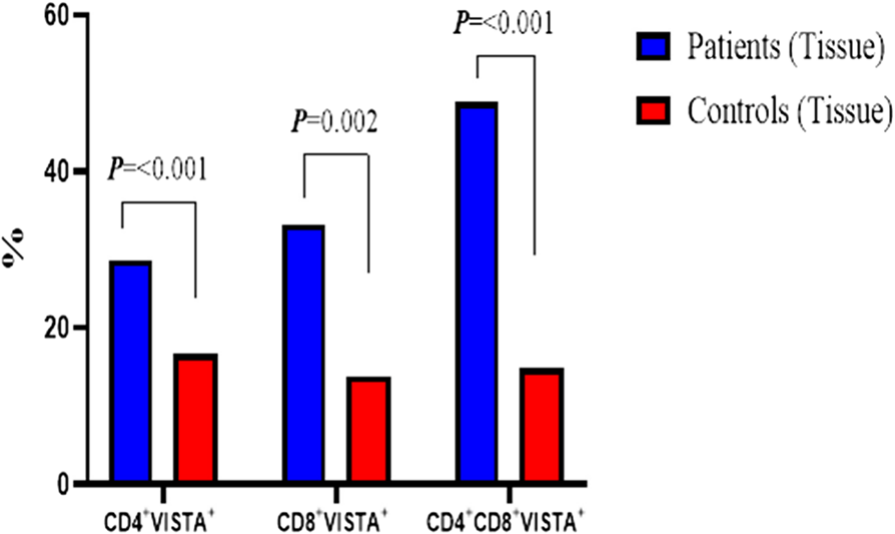
Expression of VISTA on different T cell subsets on malignant tissue and normal tissue of CRC patients

There was no significant difference in the level of CD4^+^T helper cells and the level of double positive T cells (CD4^+^CD8^+^) in malignant tissue of CRC patients and that of normal tissue. The level of CD8^+^T cytotoxic cells in the malignant tissue of CRC patients was significantly higher than that in the normal tissue. The expression of VISTA on CD4^+^T helper cells, CD8^+^T cytotoxic cells, and double positive T cells (CD4^+^CD8^+^) in the malignant tissue of CRC patients were significantly higher than that in normal tissue. As shown in Table 7 and Figs. 5, 6, 7 and 8.

**Table 6:** Correlation with some clinical and laboratory data and T cells subsets and their VISTA expression in peripheral blood

Variables	T helper cells (CD4^+^)		T cytotoxic cells (CD8^+^)		Double positive (CD4^+^CD8^+^)	
	*r*	*p*-value	*r*	*p*-value	*r*	*p*-value
Age	−0.198	0.285	0.183	0.327	0.195	0.293
NLR	−0.168	0.365	−0.121	0.517	−0.052	0.782
PLR	−0.091	0.627	−0.131	0.481	−0.162	0.384
LMR	0.108	0.563	0.049	0.796	−0.178	0.337
Hb level	−0.302*	0.024	0.123	0.368	0.029	0.833
WBC count	0.119	0.383	−0.013	0.923	−0.028	0.837
Platelet count	0.145	0.287	0.061	0.657	−0.046	0.736
urea	−0.027	0.887	−0.119	0.525	−0.026	0.891
LDH	−0.206	0.265	−0.149	0.425	−0.287	0.118
AST	−0.050	0.789	−0.199	0.283	−0.182	0.328
ALT	0.267	0.146	−0.255	0.167	−0.258	0.161
ALP	0.098	0.600	−0.228	0.218	0.018	0.923
Total protein	0.212	0.253	0.226	0.221	0.422	0.422
Albumin	0.256	0.165	0.016	0.930	−0.084	0.654
CEA	0.028	0.882	−0.032	0.864	0.293	0.110
CA19.9	−0.047	0.801	−0.011	0.952	0.313	0.086
Variables	CD4^+^VISTA^+^		CD8^+^VISTA^+^		CD4^+^CD8^+^VISTA^+^	
	*r*	*p*−value	*r*	*p*−value	*r*	*p*−value
Age	0.017	0.926	0.138	0.458	−0.079	0.672
NLR	−0.137	0.463	0.104	0.577	−0.224	0.227
PLR	−0.010	0.956	0.186	0.316	−0.295	0.107
LMR	−0.056	0.766	−0.108	0.564	0.044	0.815
Hb level	0.565	0.003	−0.114	0٫402	0.149	0.272
WBC count	0.314*	0.019	0.219	0.105	0.089	0.516
Platelet count	0.382*	0.004	0.268*	0.046	−0.030	0.826
urea	0.090	0.630	0.083	0.658	0.077	0.682
LDH	−0.260	0.158	−0.021	0.909	−0.018	0.923
AST	−0.149	0.423	−0.233	0.208	0.165	0.376
ALT	0.004	0.985	0.017	0.929	0.156	0.402
ALP	−0.144	0.440	0.009	0.962	−0.149	0.423
Total protein	0.237	0.200	0.245	0.183	0.135	0.469
Albumin	0.006	0.974	0.088	0.637	0.017	0.927
CEA	0.175	0.347	0.402	0.025	−0.091	0.626
CA19.9	0.057	0.759	0.284	0.122	−0.025	0.893
*r* correlation coefficient. *p < 0.05 is considered statistically significant analysis done by Spearman correlation. *Hb* hemoglobin, *WBC* white blood cells, *LDH* lactate dehydrogenase, *AST* aspartate transaminase, *ALT* alanine transaminase, *ALP* alkaline phosphatase, *CEA* carcinoembryonic antigen, *CA19.9* cancer antigen 19.9, *NLR* neutrophil/lymphocyte ratio, *LMR* lymphocyte/monocyte ratio, and *PLR* platelet/lymphocyte ratio

**Table 7:** T cells subsets and their Expression of VISTA in malignant tissue and normal tissue of CRC patients

Variables	Malignant tissue (*n* = 31)	Normal tissue (*n* = 25)	*p*-value
T helper (CD4^+^) cells (%)	13.55 ± 1.27	17.61 ± 0.81	0.06
CD4^+^VISTA^+^cells (%)	28.64 ± 3.13	16.62 ± 0.919	0.003
T cytotoxic (CD8^+^) cells (%)	25.93 ± 0.56	17.84 ± 0.61	< 0.001
CD8^+^VISTA^+^cells (%)	33.176 ± 3.5	13.8 ± 0.91	0.002
CD4^+^CD8^+^ T cells (%)	1.16 ± 0.09	1.0 ± 0.08	0.457
CD4^+^CD8^+^VISTA^+^cells (%)	48.92 ± 4.59	14.86 ± 0.779	< 0.001

Results are expressed as mean ± standard error of mean

Mann–Whitney *U* test, *p*-value is significant at < 0.05

As shown in Table 8, expression of VISTA on malignant tissue was significantly higher in (CD8^+^) cytotoxic T cells than (CD4^+^) T-helper cells, and its expression on (CD4^+^CD8^+^) double positive T cells was significantly higher than (CD8^+^) cytotoxic T cells. However, expression of VISTA on normal colonic tissue was not significantly different between CD4^+^T helper cells, CD8^+^T cytotoxic cells, and CD4^+^CD8^+^ double positive cells.

**Table 8:** Determine the localized expression pattern of VISTA on T cell subsets in malignant tissues of CRC patients and the normal tissues

Variables	CD4^+^VISTA^+^	CD8^+^VISTA^+^	CD4^+^CD8^+^VISTA^+^	*p*-value
Patients	28.64 ± 3.13	33.176 ± 3.5	48.92 ± 4.59	< 0.001
Normal controls	16.62 ± 0.919	13.8 ± 0.91	14.86 ± 0.779	0.105

Results are expressed as mean ± standard error of mean

Kruskal–Wallis Test, *p*-value is significant at < 0.05

#### Relation between CRC prognostic markers and T cells subsets and their VISTA expression in malignant tissue

**Table 9:** Correlation between age, NLR, PLR, and LMR data and T cells subsets and their VISTA expression in malignant colonic tissue

Variables	T helper cells (CD4^+^)		T cytotoxic cells (CD8^+^)		Double positive (CD4^+^CD8^+^)	
	*r*	*p*-value	***r***	***p*-**value	***r***	***p*-**value
Age	− 0.347	0.056	0.123	0.510	0.195	0.293
NLR	0.048	0.799	− 0.023	0.901	− 0.052	0.782
PLR	− 0.036	0.849	− 0.074	0.691	− 0.162	0.384
LMR	0.039	0.834	− 0.033	0.858	− 0.179	0.337
Variables	CD4^+^VISTA^+^		CD8^+^VISTA^+^		CD4^+^CD8^+^VISTA^+^	
	***r***	***p*** − value	***r***	***p* − **value	***r***	***p* − **value
Age	0.297	0.104	0.310	0.089	− 0.338	0.063
NLR	− 0.007	0.968	0.099	0.598	− 0.057	0.762
PLR	0.045	0.812	0.098	0.598	− 0.082	0.659
LMR	− 0.019	0.921	0.022	0.906	0.096	0.609

^*^p < 0.05 is considered statistically significant analysis done by Spearman correlation. *NLR* neutrophil/lymphocyte ratio, *PLR* platelets/lymphocyte ratio, *LMR* lymphocyte/monocyte ratio

As shown on Table 9; there were no significant correlation between T cell subsets and their VISTA expression and age, NLR and PLR data in malignant colonic tissue.

#### Relation between CRC site and T cells subsets and their VISTA expression in malignant colonic tissue

There was no significant association between site of CRC cancer and T cells subsets or their expression of VISTA on malignant tissue.

#### Relation between CRC stages and T cells subsets and their VISTA expression in malignant tissue

There was no significant association between CRC stages and T cells subsets or their expression of VISTA on malignant tissue of CRC patients.

### T cell subsets and their VISTA expression in PB, normal tissue and malignant tissue of CRC patients

As shown in Table 10, CD4^+^ T helper cells showed significantly decrease in malignant tissue of CRC patients and normal tissue than PB. In addition to, CD8^+^ T cytotoxic cells was significant higher in malignant tissue than normal tissue and PB. Double positive T cells showed no significant difference in malignant tissue, normal tissue, and PB.

**Table 10:** Different expression of T cells subsets and their VISTA expression on malignant tissue, normal tissue and PB of CRC patients

Variables	Malignant tissue	Normal tissue	Peripheral blood	*p* − value
T helper (CD4^+^) cells (%)	13.87 ± 0.449	17.619 ± 0.813	31.56 ± 1.277	< 0.001
CD4^+^VISTA^+^cells (%)	28.64 ± 3.13	16.62 ± 0.919	13.24 ± 2.625	< 0.001
T cytotoxic (CD8^+^) cells (%)	25.93 ± 0.97	17.82 ± 0.240	23.78 ± 1.32	0.001
CD8^+^VISTA^+^cells (%)	33.176 ± 3.59	13.816 ± 0.912	15.41 ± 2.22	< 0.001
CD4^+^CD8^+^ T cells (%)	1.16 ± 0.109	1.08 ± 0.12	1.1 ± 0.108	0.995
CD4^+^CD8^+^VISTA^+^cells (%)	48.92 ± 4.59	14.86 ± 0.77	15.15 ± 2.344	< 0.001

Results are expressed as mean± standard error of mean

Kruskal–Wallis test,* p*-value is significant at < 0.05

The expression of VISTA on CD4^+^T helper, CD8^+^T cytotoxic, and double positive T cells (CD4^+^CD8^+^) on malignant tissue was significantly higher than its expression on CRC patient’s PB and normal tissue.

## Discussion

Colorectal cancer (CRC) is one of the pervasive tumors in the world were more than 1.2 million cases of CRC are diagnosed each year and it is the third most common cause of cancer that affects humans [1, 2]***.*** It is considered the second and third most commonly diagnosed cancer in females and males, respectively. It is caused by mutations that affect oncogenes, tumor suppressor genes, and genes involved in DNA repair processes in the epithelial tissues of the colon and rectal regions [28].

In this study, there was a male predominance in CRC patients which may be due to the differences in biological and gender behavioral factors. Mean age 52.71 ± 2.390, thus most are middle to old aged, which indicate the risk of CRC increases with advancing age. As reported in multiple previous studies, [29–40].

In concordance with previous studies like Am et al. [41], only about one third of CRC patients were smokers which may indicate the lack of association of CRC with smoking. Most patients in the present study presented with bowel habit change, rectal bleeding, abdominal pain, and weight loss. Also similar to previous study of de Sousa et al. [42], most of their patients presented with change in bowel habitat, rectal bleeding, abdominal pain, and weight loss. Also, Nisar et al. [43] found that most CRC patients presented with bleeding per rectum, anemia, weight loss, and altered bowel habits.

In this study there was a significant increase of platelets count, total leukocytes count, neutrophils, and monocytes in CRC patients than the normal controls and significant decrease in lymphocytes than the normal controls. This finding may indicate of bone marrow reaction as a response to CRC. Platelets can coat tumor cells, keeping them invisible to the immune system’s natural killer cells, they have been identified as promoters of metastases. Furthermore, platelets affect signaling pathways of DNA repair by the activation of epidermal growth factor receptor (EGFR) and DNA − dependent protein kinase. Platelet − derived growth factor (PDGF) is also a key factor in tumor growth and invasion. Additionally, cytokines and chemokines which are produced by platelets, may contribute to the development of cancer − related inflammation [44]. Additionally, tumor cells themselves can modify platelet activity to best control tumor growth, proliferation, metastasis, and survival [45, 46].

Neutrophils promote cancer cell proliferation, angiogenesis, and metastasis by the production of proangiogenic chemokines and growth factors such as vascular endothelial growth factor (VEGF***)*** [47]. Monocytes seem to be enlisted as inflammatory cells that possess the biological apparatus necessary to eliminate cancerous cells directly. M1 and M2 are the two phenotypes linked to monocyte activity in cancer. The anti − cancer response is enhanced by M1 macrophages through the production of IL − 1, IL − 6, IL − 12, TNF − α, ROS, and RNI [48]. The production of IL − 12, IL − 23, and IL − 10 by cells with the M2 phenotype helps to kill inflammation and reduce the body’s anti − cancer immune response.

Colorectal cancer (CRC) exhibits the highest correlation between the development of cancer and chronic inflammation. When utilized separately as predictive indicators for individuals with colorectal cancer, the ratios obtained from the commonly tested inflammatory biomarkers exhibit poor performance and limited clinical usefulness [49].

In our study we found, significant increase of NLR and PLR in CRC patients than the normal controls and decrease in LMR in CRC patients than the normal controls. Higher NLR values may indicate an imbalance between neutrophils and lymphocytes may be linked to the development of cancer. High PLR values in CRC patients than the normal controls indicate to increase platelets count and decrease in lymphocytes count in CRC patients than the normal controls. Elevated PLR are linked to increase tumor aggression and metastasis and correlate with advanced stages of CRC [50]. The LMR in CRC patients has emerged as a potential prognostic marker, as it can reflect the balance between the immune system’s response to the tumor and inflammation within the body and may indicate a reflecting an inflammatory environment that promotes tumor progression.

Our results agreed with Li et al. [50] and Choi et al. [37], who showed that CRC patients had significantly higher in total leukocytes count, platelets count, NLR, and PLR. Also, [51] found that white blood cell, neutrophil and NLR in patients significantly higher than normal controls. And matching to reports of Kurt et al. [52]***,*** who found that a rise in circulating neutrophils and a corresponding drop in circulating lymphocytes during the carcinogenesis process.

Most of CRC patients with low level of RBCs count and HB level than the normal controls which may be due to sever rectal bleeding which associated with the CRC. Which agreed with Li et al. [37] found that HGB and RBC counts were significantly lower in CRC patients than the normal controls.

In this study, there were significantly increase in the levels of urea, aspartate transaminase (AST), alkaline phosphatase (ALP), lactate dehydrogenase (LDH), and random blood glucose (RBG) in CRC patients than normal controls. Increased glucose levels in CRC patients might indicate cancer − related cachexia, insulin resistance, or tumor − induced metabolic changes. Some tumors, including CRC, can alter metabolic pathways and increase glucose uptake. Increased levels of LDH in CRC patients may reflect tumor cell turnover (rapid cell death and regeneration), tumor progression, or metastasis. It is often used as a marker of tissue damage and can indicate poor prognosis in cancer, particularly when associated with advanced or metastatic disease. Chronic inflammation in CRC patients can elevate various biomarkers, as systemic inflammatory responses can affect liver and kidney function, glucose metabolism, and tissue damage. CRC and other cancers can disrupt normal metabolism, leading to altered levels of these biomarkers.

In accordance with, Addissouky et al. [53] and Almehmadi [54] found that AST and ALP was significantly increase in CRC patients than the normal controls. Chung et al. [55] and Tsushima et al. [56] found that RBG in CRC patients was significantly higher than the normal controls.

Our result in contrast with Li et al. [37], who detected that AST and ALT were significantly decrease in CRC patients than the normal controls and Kostakis et al. [57] found LDH levels have no significant difference in CRC patients and the normal controls.

Most of patients (74.19%) had moderately differentiated adenocarcinoma and 25.8% had mucinous adenocarcinoma. Our results are more or less similar to the results of Fazeli et al. [58] who found that adenocarcinoma was seen in 96.2% of the patients. Also, Am et al. [41] who found that the predominant subtype of CRC was adenocarcinoma (70.7%) patients, followed by mucinous differentiation in (16.7%) patients, signet ring carcinoma in (11.4%) patients, and squamous cell differentiation in (1.1%) patients.

The study showed that 67.6% patients had “T3” followed by (25.8%) patients had “T2,” and (6.5%) patients at “T4.” Lymph node involvement, each “N0” and “N1” were evident in (41.9%) patients, while (16.13%) patients exhibited “N2.” Concerning metastasis, (77.4%) patients were at “M0” and (22.58%) patients were at “M1.” These results are agreed with results of [33, 34, 50, 59]***.***

Stage (III) was the most common followed by stage (II), stage (I), and finally stage (IV). Our results agreed with NISAR, Faizi, and Ali 2016, who found that (41.90%) of CRC patients presented with stage III, (31.43%) with stage II and (26.67%) patients with stage I. Chen et al. 2022, found that (36.81%) stage (II) followed by (34.3%) stage (III), (15.7%) stage (IV) and finally stage (I) in (13.19%) patients.

In this study, all cases had “grade 2” tumors (100%). This is in opposing to Savu et al. [30], Savu et al. [33], Diez-Alonso et al. [34], Ulanja et al. [60], and Negri et al. [61], where their studies showed grade 1, grade 2, and grade 3, and this disagreement is mostly due to the small number of cases in our study.

RT colon cancer was detected in 35.48%, and LT colon cancer in 64.56% patients. This result is concordance with [31, 36, 61]***.***

There was significant decrease in the level of CD4^+^T helper cells and increase in the level of CD8^+^T cytotoxic cells in CRC patients than the normal controls. In CRC, a reduction in Th cells is often associated with immune evasion, tumor progression, and poor prognosis. The possible explanation for the reduction of CD4^+^T helper in CRC patients is T helper cells, due to its critical role in orchestrating the immune response against tumors and for their ability to induce effective anti − tumor responses by producing pro − inflammatory cytokines such as IFN − γ. However, CD8^+^ T cells were present at higher frequencies in the PB of CRC patients as part of the body’s immune response to tumor cells. This increase in CD8^+^ T cells reflects an attempt by the immune system to mount an effective response against the growing tumor. Our results are in agreement with Attallah et al. [62], Spacek et al. [63], Waidhauser et al. [64], and Zhang et al. [65], who found decrease in the levels of helper T lymphocytes (CD4^+^) in CRC patients’ comparison to the normal controls. Hu et al. [66] found that the percentages of CD8^+^ T cells was increased in the peripheral blood with peritoneal neoplasms patients compared with the normal controls.

In contrary to our results, Krijgsman et al. [67] and Choi et al. [51] found no significant difference in the distribution of circulating CD8^+^ and CD4^+^T lymphocytes in the PB of CRC patients and normal controls. Also, Riazi Rad et al. [68] and Schröder et al. [69] found that there were not any significant differences in CD4^+^ and CD8^+^ T cells of the PB between the breast cancer (BC) patients and the normal controls.

There was significant increase in expression of VISTA on both CD4^+^T helper and CD8^+^ T cytotoxic cells of CRC patients than in the normal controls. Higher expression of VISTA in patients with CRC may contribute to T cell exhaustion and a damaged anti − tumor response, and may suggest that VISTA may be considered a potential target for reversing T cell exhaustion and improving T cell function in CRC patients.

To the best of our knowledge there was no previous study of VISTA expression on T cell subsets of CRC patients. However, there is some previous studies in different types of cancer***.*** Huang et al. [70] found that there were significantly increased of VISTA expression on CD4^+^ T helper and CD8^+^ T cytotoxic in multiple myeloma patients, compared to the normal controls. Also, Wang et al. [71] found that VISTA + T cells was increased in the CD4^+^ T and CD8^+^ T − cell subset in the PB of patients with AL amyloidosis and normal controls.

In addition, there were other inhibitory immune checkpoints were studied in CRC, Choi et al. [51] found that individuals with CRC had increased levels of PD1, CATLA − 4 and LAG − 3. Hu et al. [66] found that PD − 1 and Tim − 3 exhibited on circulating lymphocyte, CD4 ^+^ T cells, and CD8 ^+^ T cells and the elevated percentages of CD4 ^+^ PD − 1 T cells and CD8 ^+^ PD − 1 T cells in PB of patients with peritoneal neoplasms were significantly higher than normal controls.

Expression of VISTA on PB of CRC patients was not significantly different across three subsets of T cells: CD4 + T helper cells, CD8 + T cytotoxic cells, and CD4 + CD8 + double − positive cells. However, expression of VISTA on PB on the normal controls was Significant increase on (CD4^+^CD8^+^) double positive T cells than on (CD4^+^) T − helper cells, and on (CD8^+^) cytotoxic T cells. That may suggest that VISTA may play a generalized role in immune suppression, acting across multiple T cell subsets to regulate immune responses. This could be part of an immune evasion strategy employed by the tumor to maintain an immunosuppressive environment.

CD4^+^T helper cells showed significantly decrease in malignant tissue of CRC patients and normal tissue than PB. In addition to, CD8^+^T cytotoxic cells was significantly higher in malignant tissue than normal tissue and PB. Double positive T cells showed no significant difference in malignant tissue, normal tissue and PB. According to our findings, patients with CRC had compromised T cell − mediated immunity, which was more pronounced in the tumor tissue microenvironment than in the peripheral blood. The antitumor immunity is mediated by CD8^+^T lymphocytes. CRC patients’ “ImmunoScore,” or the density of cytotoxic CD8^+^T lymphocytes within the tumor and invasive border of a tumor sample, has become a predictive indicator.

In accordance with, Li et al. [72] who found that there were no significant changes in CD4^+^ T helper cells, between malignant colonic tissue and normal tissue. Also, Syed Khaja et al. [73] found CD8^+^T cytotoxic cells increased significantly in breast cancer tumor tissue compared with normal tissue. This is in opposing to Toor et al. [74], who found that malignant CRC tissues had a much higher concentration of CD4^+^ T lymphocytes than normal controls. According to the data of Wu et al. [75], there were significantly higher in CD8^+^T cytotoxic cells in the tumor tissue than in the peripheral blood of patients with cervical cancer, and more CD4^+^T helper cells in peripheral blood than malignant tissue.

The expression of VISTA protein was seen in both tumor cells and endothelial cells, in addition to tumor − infiltrating immune cells [26]***.*** VISTA expression is also observed on T cells inside specific tumor types [21, 76, 77]. So, the higher expression of VISTA in all tumor infiltrated T cells (CD4^+^T helper cells, CD8^+^T cytotoxic cells and double positive T cells than its expression on normal colonic tissue, and CRC patient’s PB could suggest its role in pathogenesis of CRC and VISTA may reduce T cell function in the CRC tumor microenvironment. However, previous studies discuss the expression of VISTA on malignant tissue of CRC and its expression on T cells in other types of cancer. To the best of our knowledge there was no previous study about expression of VISTA on T cell subsets on PB, malignant tissue, and normal tissue of CRC patients***.*** Elashi et al. [78] found that, in comparison to PB, the tumor tissues of CRC and BC exhibit significantly different expression of VISTA than do the circulation and normal tissues of the same CRC and BC patients. Xie et al. [21] found that VISTA is expressed in tumors and normal colorectal tissues in CRC patients, with higher expression on tumor tissue. Ma et al. [79] found that their significant increase in CD4^+^VISTA^+^T lymphocytes in non − small cell lung cancer (NSCLC) tumor tissues when compared to peritumor tissues. Also, Zhang et al. [22] found that VISTA expression was detected in hepatocellular carcinoma (HCC) tissues. In addition to, Digomann et al. [80] found that VISTA was abundantly expressed on immune cells that had infiltrated tumors in pancreatic ductal adenocarcinoma (PDAC) patients.

Xie et al. (2020) found that BC tissue expresses more of the immunological checkpoint VISTA than nearby normal tissue. VISTA expression was detected in CD8^+^ cytotoxic T cells and CD4^+^ T cells. Also, Mulati et al. [24] found that endometrial cancer patients expressed VISTA on CD8^+^ cytotoxic T cells. Also, Luk et al. [81], Digomann et al. [80], Mulati et al. [24], and Wu et al. [23] found that VISTA is highly expressed on malignant tissues than the normal tissues when examen the expression of VISTA on endothelial cells within a subset of cancers, synovial sarcoma, immune − privileged tissues, human PDAC, endometrioid, and clear cell subtypes, ovarian cancer and primary oral squamous cell carcinoma, respectively.

There was a previous study for other types of ICIs as Ahmadzadeh et al. [82] found that PD − 1 + CD8 + cytotoxic and PD − 1 + CD4 + helper T cells were higher in melanoma patient tumors than in PB from the same patients or healthy volunteers. Also, Wu et al. [83] who found that PD − 1 expression on CD8 + T lymphocytes is significantly higher in tumors and normal lymph nodes than in peripheral blood.

Expression of VISTA on malignant tissue was significantly higher in (CD8^+^) cytotoxic T cells than on (CD4^+^) T − helper cells, and its expression on (CD4^+^CD8^+^) double positive T cells significantly on (CD8^+^) cytotoxic T cells. However, expression of VISTA on normal colonic tissue was not significantly different between CD4^+^T helper cells, CD8^+^T cytotoxic cells, and CD4^+^CD8^+^ double positive cells.

The differential expression of VISTA on CD8 + cytotoxic T cells, CD4 + T helper cells, and CD4 + CD8 + double − positive T cells in malignant tissue likely reflects the tumor’s strategy of immune evasion. By upregulating VISTA specifically on CD8 + T cells, the tumor can limit the effectiveness of the primary immune effectors responsible for tumor killing. Additionally, the expression on CD4 + CD8 + double − positive cells suggests that these cells may also be part of the tumor’s regulatory network, further contributing to immune suppression. The higher VISTA expression on CD8 + cytotoxic T cells and CD4 + CD8 + double − positive cells is consistent with the tumor’s need to suppress all potential avenues of immune attack, while leaving CD4 + helper cells relatively less suppressed. These dynamic highlights the complex interplay between immune checkpoints and tumor immune evasion strategies.

Our result in agreement with Villarroel-Espindola et al. [77] who determine the localized expression pattern and clinical significance of VISTA in human NSCLC, found that VISTA expression was greater in cytotoxic T cells than in T − helper cells.

In our study, we did not find any significant relation between the expression of VISTA on T cell subsets and WBCs, laterality, gender, age, tumor stage, lymph node metastases (N − category), or distant metastases (M − category). The small sample size may have limited the capacity of detecting the potential difference. Alternatively, modulation of VISTA alone might not be dominant enough to directly dictate the clinical outcome.

In accordance with Xie et al. [21] who found that in CRC patients, tumor size, gender, age, tumor stage, lymph node metastases, or distant metastases were not correlated with VISTA expression. And agreed with Zong et al. [84] who found that there were any significant differences between VISTA expression in patients with breast cancer and cancer stage, and agreed with, Wang et al. [20] who found that there was no significant difference between the expression of VISTA and age, gender and WBC in AML patients. Mulati et al. [24] found that there was no discernible variation in VISTA expression between the ovarian tumors’ original and metastatic locations. Xie et al. [85] found that Pathological grade and lymph node status were strongly connected with VISTA expression in breast cancer tissue.

To the best of our knowledge, there was no previous study make correlation between VISTA and CEA and CA19.9. We found that there was no significant difference in the level of CEA, CA19.9, and T cells subsets in CRC patient’s peripheral blood. Also, there was no significant difference between CEA, CA19.9, and VISTA expression on CD4+ T helper cells and double positive T cells with VISTA expression on CRC patient’s PB. Also, there was significant difference between CEA, CA19.9 and VISTA expression on CD8+ T cytotoxic cells. However, there was no significant difference in the level of urea, LDH, AST, ALT, ALP, Total protein and albumin, and T cells subsets and their VISTA expression.

At other inhibitory immune checkpoint level, Elashi et al. [78] found that groups of patients with CRC and BC were compared according to their TNM staging and histological grade. The expressions of immunological checkpoints (PD− 1, CTLA− 4, TIM− 3, LAG− 3, TIGIT, PD−L1, and VISTA) did not significantly differ across any categories, according to their findings.

## Conclusion

The higher expression of VISTA in CRC patients than the healthy controls and its higher levels in malignant CRC tissue and normal colonic tissue than PB of CRC patients suggest the role VISTA in the pathogenesis of CRC.

## Data Availability

The datasets generated and/or analyzed during the current study are available from the corresponding author on reasonable request. The authors declare no competing interests.

## Abbreviations

ALT: Alanine transaminase
APC: Allophycocyanin
AST: Aspartate transaminase
BTLA: B and T lymphocyte attenuator
CA19.9: Cancer antigen 19.9
CEA: Carcinoembryonic antigen
CRC: Colorectal cancer
CBC: Complete blood count
CTLA- 4: Cytotoxic T lymphocyte associated molecule-4
DP: Double positive T cells
ICs: Immune cells
INR: International Normalized Ratio
LDH: Lactate dehydrogenase
M: Metastasis
NLR: Neutrophil/lymphocyte ratio
Percp: Peridinin-chlorophyll-protein
PB: Peripheral blood
PBS: Phosphate buffer saline solution
PE-Cy7: Phycoerythrin
PLR: Platelet/lymphocyte ratio
PD- 1: Programmed death-1
RBG: Random blood glucose
SECI: South Egypt Cancer Institute
SE: Standard error
Tim- 3: T cell immunoglobulin mucindomain-containing-3
LAG- 3: T cell lymphocyte activation gene-3
TLC: Total leukocytes count
TILs: Tumor-infiltrating lymphocytes
VISTA: V-domain immunoglobulin suppressor of T cell activation
WHO: World Health Organization
